# New-generation biofilm effective antimicrobial peptides and a real-time anti-biofilm activity assay: CoMIC

**DOI:** 10.1007/s00253-024-13134-1

**Published:** 2024-05-03

**Authors:** Tuba Polat, İrem Soyhan, Sinan Cebeci, Tuğba Arzu Özal İldeniz, Özgül Gök, Merve Açıkel Elmas, Erkan Mozioğlu, Nihan Ünübol

**Affiliations:** 1 Department of Medical Biotechnology, Institute of Health Sciences, Acibadem Mehmet Ali Aydinlar University, Istanbul, Turkey; 2 Department of Biomedical Engineering, Faculty of Engineering and Natural Sciences, Acibadem Mehmet Ali Aydinlar University, Istanbul, Turkey; 3 Department of Medical Microbiology, School of Medicine, Acibadem Mehmet Ali Aydinlar University, Istanbul, Turkey; 4 Department of Histology and Embriology, School of Medicine, Acibadem Mehmet Ali Aydinlar University, Istanbul, Turkey; 5 Medical Laboratory Techniques, Vocational School of Health Services, Acibadem Mehmet Ali Aydinlar University, Istanbul, Turkey

**Keywords:** Anticrobial peptides, Biofilm, Anti-biofilm Activity Assay, Protease resistant

## Abstract

**Abstract:**

Nowadays, it is very important to produce new-generation drugs with antimicrobial properties that will target biofilm-induced infections. The first target for combating these microorganisms, which are the source itself. Antimicrobial peptides, which are more effective than antibiotics due to their ability to kill microorganisms and use a different metabolic pathway, are among the new options today. The aim of this study is to develop new-generation antibiotics that inhibit both biofilm-producing bacteria and the biofilm itself. For this purpose, we designed four different peptides by combining two amino acid forms (D- and L-) with the same sequence having alpha helix structures. It was found that the combined use of these two forms can increase antimicrobial efficacy more than 30-fold. These results are supported by molecular modeling and scanning electron microscopy (SEM), at the same time cytotoxicity (IC_50_) and hemotoxicity (HC_50_) values remained within the safe range. Furthermore, antibiofilm activities of these peptides were investigated. Since the existing biofilm inhibition methods in the literature do not technically simulate the exact situation, in this study, we have developed a real-time observable biofilm model and a new detection method based on it, which we call the CoMIC method*.* Findings have shown that the NET1 peptide with D-leucine amino acid in its structure and the NET3 peptide with D-arginine amino acid in its structure are effective in inhibiting biofilm. As a conclusion, our peptides can be considered as potential next-generation broad-spectrum antibiotic molecule/drug candidates that might be used in biofilm and clinical important bacteria.

**Key points:**

*• Antimicrobial peptides were developed to inhibit both biofilms producing bacteria and the biofilm itself.*

*• CoMIC will fill a very crucial gap in understanding biofilms and conducting the necessary quantitative studies.*

*• Molecular modelling studies, NET1 peptide molecules tends to move towards and adhere to the membrane within nanoseconds.*

**Supplementary Information:**

The online version contains supplementary material available at 10.1007/s00253-024-13134-1.

## Introduction

Microorganisms have evolved over many years and developed mechanisms that cause infections by both protecting from environmental effects and surviving on humans (Ganz and Lehrer 1999). In recent years, scientists have suggested peptide antibiotics, produced by organisms living in nature, as an alternative to existing antibiotics, and started a new era in humanity's war against microorganisms. Peptide antibiotics are molecules in peptide structure produced by organisms to protect themselves against infections. These are produced by the relevant organisms (plant, animal, or microorganisms, etc.). For the treatment of resistant microorganisms that develop in hospitals, it is important to modify and develop existing natural peptide antibiotics to have broad-spectrum effects. In this context, many studies have shown that molecules inspired by natural peptide antibiotics and artificially produced can be used. Many studies have also shown that peptide antibiotics are suitable for the production of new antimicrobial drugs, since they are natural molecules, have low toxicity to human cells and have broad-spectrum activity on microorganisms (Brogden and Brogden 2011; Unubol et al. 2017).

The fact that these new nanotechnological products, which make humanity advantageous against sole life forms of microorganisms, can also be effective against biofilms, which are their much more resistant structures, stands before us as another requirement.

Biofilm is a structure produced by microorganisms, consisting of polymer materials, adhering to living or non-living surfaces, enabling the microorganism to adapt to its new environment and protecting it against the effects of the external environment and making it resistant (Nguyen et al. 2011; Mookherjee et al. 2020). Microorganisms adhere to their environment by producing a biofilm and are protected from the antimicrobial effect of antibiotics and phagocytosis (Hall-Stoodley et al. 2004; López et al. 2010). The biofilm layer is an extremely important structure in providing resistance against the effects of environmental factors and antimicrobial substances that will harm the microorganism. There is a need for new-generation antibiotics with a broad spectrum and long-lasting antimicrobial and antibiofilm activity especially due to the increase in patients receiving long-term treatment in hospitals. Thanks to the properties of peptide antibiotics, the development of new drugs with antimicrobial properties stands out as a strong option (Brogden and Brogden 2011). However, considering that secreted microbial proteases have an important role in the formation and dispersion of biofilms, it is crucial that the new peptide antibiotics slade should be resistant to proteases.

For this purpose, here, we designed protease resistant four peptide antibiotics by combining two amino acid forms (D- and L-). Then, we tested their antimicrobial/antibiofilm efficacies by in vitro MIC assay. Cytotoxicity (IC_50_ which is the cytotoxic concentration lysing 50% of cells), and hemotoxicity (HC_50_ which is the hemolytic concentration lysing 50% of cells) assays were also performed. Furthermore, the results were supported by molecular modeling and SEM.

Many different methods are used to test for new antibiofilm agents. These are classified as direct and indirect methods such as microscopy (light microscopy, FISH, confocal microscopy, TEM, SEM, AFM), the roll plate method, mass spectrometry, QCM-based method, impedance-based method, Raman spectroscopy, Congo Red agar method, crystal violet method in plates/tubes, PCR, and nuclease-based method (Kırmusaoğlu 2019; Mozioğlu and Kocagöz 2020; Goudarzi et al. 2021; Slade et al. 2022; Kamimura et al. 2022; Salazar et al. 2023; Kumar et al. 2023). Some of them (such as PCR, SEM, TEM, mass spectrometry, AFM, Raman, QCM and impedance-based methods) require sophisticated expensive instrumentation and are not suitable for high throughput studies, especially for the study of multiple antibiofilm agents at different concentrations, while others (crystal violet and Congo Red agar methods) except nuclease-based method (Mozioğlu and Kocagöz 2020) are easy to perform but semi-quantitative methods (Slobbe et al. 2009).

One of the most widely used methods in literature in terms of being easy, fast, and cheap is crystal violet method. Although it is highly suitable for multiple studies for determining the minimal inhibitory concentration (MIC) for biofilms in liquid cultures, inconsistencies are sometimes experienced between the results obtained with this end point method (Castro et al. 2022). Another commonly used method is Congo Red agar. In this method, biofilm producing microorganisms can be detected as black colored colonies on red agar medium. As an alternative to this end point agar method, a limited number of studies on Congo Red broth have also been published, in which black color transformation is observed at the end of biofilm culture (HRV et al. 2016). Unfortunately, these end point methods have major limitations as there is no other way to test for antibiofilm agents than adding them at the very beginning of the biofilm culture (Thieme et al. 2019). To overcome this current problem of biofilm methodology in literature, new methods are needed to determine the point at which biofilm formation begins. Unlike the method based on the binding of crystal violet to the extracellular matrix forming the biofilm, Congo Red provides black color formation due to some components during biofilm formation. This gives us the opportunity for a cheap, simple and fast way to detect when biofilm formation begins as well as to monitor all biofilm formation in real time. Based on this principle, in this study, we developed a method to enable real-time monitoring of biofilms by spectrophotometric measurement of the red-to-black conversion of Congo Red broth, we call CoMIC method (Congo red-minimal inhibitory concentration (MIC) for biofilms) (Thieme et al. 2019).

## Materials and methods

### Design of peptide antibiotics, molecular modeling, and molecular dynamics studies

It is well-known that antimicrobial peptides are commonly hydrophobic and positively charged in the literature (Breij et al. 2018). In our study, peptides with a length of 10 to 20 amino acids, which form an α-helix, similar to the structure of LL-37, were designed with hydrophobic and positively charged amino acids. The D-amino acid form is also used in the design of peptide antibiotics. The use of D-amino acid not only makes the peptide more stable, but also provides resistance to proteases (Hamamoto et al. 2002; Manabe and Kawasaki 2017). The amide group at the C-terminal end causes the peptide to approach perpendicular to the membrane and to be taken up into the cell more rapidly. It is very important for the peptides to terminate with an amide group, as this affects the increase in membrane permeability. It is also thought to have an effect on resistance to proteases (Jo et al. 2008). Four peptides were designed using D- and L-form amino acids in this study within the scope of all this information (Table 1).

**Table 1:** Designed peptide sequences and their properties

	Peptide sequences	Amino acid content	Hydrophobicity rate %	Net charge	pI value	Molecular weight g/mol
NET1	RLLLRLLRRLLRLLLR-NH_2_	D- leucine, L- arginine	62.5	+7	13.2	2085.75
NET2	RLLLRLLRRLLRLLLR-NH_2_	L- leucine, L- arginine	62.5	+7	13.2	2085.75
NET3	RLLLRLLRRLLRLLLR-NH_2_	L- leucine, D- arginine	62.5	+7	13.2	2085.75
NET4	RLLLRLLRRLLRLLLR-NH_2_	D- leucine, D-arginine	62.5	+7	13.2	2085.75

The 3D structures of the designed new peptides were obtained using the PEP-FOLD3 server (Thevenet et al. 2012) and molecular dynamics models were performed by running the CHARMM force field parameters and NAMD 2.11 software in parallel (Phillips et al. 2005). The placement of peptide molecules on the POPE membrane at a certain distance was done using Visual Molecular Dynamics (VMD) software (Humphrey et al. 1996). The reason for choosing the POPE membrane is that PE (Phosphatidylethanolamine) lipids are generally more abundant on the cytoplasmic side of the bacterial membrane and PE lipids are frequently used to mimic the bacterial membrane (Van Meer et al. 2008).

### Solid-phase chemical synthesis and HPLC analyses of AMPs

CEM Liberty Blue peptide synthesizer was used for peptide synthesis. At the end of the synthesis, the peptides were cut with the Razor device as stated in the manual. The synthesized peptide antibiotics were first prepared at 2 mg/ml for reversed phase high-performance liquid chromatography (HPLC) and run on the analytical column and their approximate hydrophobicity was calculated according to the peak position. Afterwards, 10 mg/mL was prepared and run in the semi-preparative C-18 column and pure peaks were collected. Collected samples were lyophilized. Concentration measurements of antimicrobial peptides (AMPs) obtained in lyophilized form were made using the Pierce Quantitative Fluorometric Peptide Assay (Thermo Fisher) kit.

### Protease experiments

Proteinase K was used for testing the resistance of the designed peptides to proteases, since Proteinase K, a serine protease with broad-spectrum cleavage activity that preferentially cleaves aliphatic and aromatic amino acid residues, is widely used in the literature to determine the protease resistance of antimicrobial peptides (Lucio et al. 2017, Wang et al. 2019). Afterwards, HPLC characterization was performed to understand the effect of Proteinase K on peptides.

### Minimum inhibitory concentration assay (MIC)

NET1, NET2, NET3, and NET4 peptide antibiotics were used in the experiments. For the purpose of the study, both clinical isolates Methicillin-Resistant *S. aureus* (MRSA), Vancomycin-resistant *Enterococcus* (VRE), *E. coli* ER2566, *E. coli* 13846 bacteria and ATCC strains *S. aureus* and *E. coli* bacteria were used in the MIC study. All strains used in this study have been deposited in a strain collection of the Research Laboratory of Acibadem Mehmet Ali Aydinlar University. For experiment 0.5 McFarland bacterial suspension was prepared in Müeller Hinton Broth (MHB) media. In the 96-well plate, serial dilutions of peptide antibiotics starting from 512 to 0 μg/mL were made in MHB media and added 5 µl of bacterial suspensions. After incubated overnight at 37°C, results were evaluated according to turbidity (Teh et al. 2017). Ampicillin was used as positive controls. The MIC value of each peptide is given in Table 2.

**Table 2:** MIC values of NET1, NET2, NET3, and NET4 peptides

(µg/mL)	NET1	NET2	NET3	NET4
*E. coli ATCC25922*	2	64	4	8
*S. aureus ATCC25923*	4	32	2	16
*MRSA*	2	64	2	16
*S. aureus ATCC29213*	2	64	2	16
*E. coli NTCC13846*	4	64	2	32
*E. coli ER2566*	<0.25	4	0.5	1
*VRE*	0.5	2	0.5	2

### Hemolytic activity

Hemolytic activity assays were carried out using erythrocytes, using triton X-100 as lysis control agent. Hemolytic activities were analyzed by reading each well with a plate reader spectrophotometer device at 414 nm and the % lysis rate was calculated in Microsoft Excel according to the following formula (Eren et al. 2008).

$$\text{Lysis }\%=\lbrack{\mathrm{OD}}_{414}-{\mathrm{OD}}_{414}\text{blank}\rbrack/\lbrack{\mathrm{OD}}_{414}\mathrm{total\,lysis}-{\mathrm{OD}}_{414}\text{blank}\rbrack\times100$$

### Cytotoxicity assay

MTT Cell Proliferation Kit (Sigma), a colorimetric method, was used for this test to evaluate the damage that peptides can cause in eukaryotic cells and all cell lines used in this study have been deposited in a collection of the Research Laboratory of Acibadem Mehmet Ali Aydinlar University. The cell lines of 3T3 (ATCC CRL-1658) and HaCaT (ATCC PCS-200-011) were revived under appropriate culture conditions and made ready for the experiment. The final concentration of peptides reached 64 to 0.5 μg/mL. Samples were worked in triplicates for each concentration. Cells incubated without peptide-treatment under the same conditions were used as a control. Magainin II, a known natural AMP that has no cytotoxic effects, was used as a positive control (Bacalum and Radu 2015). The results were analyzed by reading them with a 96-well plate reader at 550 and 690 nm.

### CoMIC method as antibiofilm activity assay

#### Optimization of different bacterial strains and sugar sources

Three different Congo Red Broth media containing 0.08% Congo Red and different sugar sources (8% glucose / 5% sucrose / 4% fructose) in Mueller Hinton Broth were used for optimization studies. There was 195 µL of culture media transferred into wells and 5 µL of 0.5 McFarland bacteria such as *Enterococcus faecalis* ATCC 29212, *S. aureus* ATCC 25923, *S. aureus* MRSA, *Klebsiella pneumoniae* ATCC 700603, *Pseudomonas aeruginosa* ATCC 27853, and *E. coli* NTCC 13846. All bacteria except *P. aeuroginosa* ATCC 27853 used here were well known to produce biofilms. *P. aeuroginosa* ATCC 27853 (Perez et al. 2011) and culture without bacteria were used as negative control for returning to black color. All cultures were performed as triplicate and incubated at 37°C overnight and fluorescence measurements were conducted by using Varioscan (Thermo, USA). The excitation/emission wavelengths were used as 525/625 nm.

#### Fluorescence changes as a function of bacterial density

The relationship between the number of bacteria and the black color change resulting in fluorescence signal change was investigated. For this purpose, 1:2 serial dilutions (0.5–0 McF) of *S. aureus* 29213 were prepared in Congo Red Broth media with sucrose. 100 µL of these dilutions were transferred into 96 well plates. All cultures were performed as triplicate and incubated at 37°C overnight and fluorescence measurements were conducted by using Varioscan (Thermo, USA). The excitation/emission wavelengths were used as 525/625 nm. As a replica representing the same conditions, bacteria in MHB media without Congo Red were cultured and spectrophotometrically monitored in real time by Varioscan (Thermo, USA) at 600 nm.

#### Real-time monitoring with CoMIC method of antibiofilm activities

1:2 serial dilutions of *ciprofloxacine (200****–****0 µg/mL) and sodium hypochlorite (5****–****0%)* were prepared in Congo Red Broth media with sucrose. *S. aureus* 29213 were added into culture media with a final concentration of 1×10^6^ Colony-forming unit/mL (CFU/mL). All cultures were performed as triplicate and incubated at 37°C overnight and fluorescence measurements were conducted by using Varioscan (Thermo, USA). The excitation/emission wavelengths were used as 525/625 nm. As a replica plate, bacteria in the same broth media, except Congo Red were cultured and analyzed with conventional Crystal Violet (CV) method. For this purpose, the bacterial cultures were removed, and wells were washed with distilled water. There was 200 µl of 1% CV dye added to each well and incubated at room temperature for 15 min in the dark. Then, CV dye was removed, and wells were washed with water. After the plate was dry, 33% acetic acid was added into wells to dissolve the dye and wells were spectrophotometrically read by Varioscan (Thermo, USA) at 590 nm (Stepanović et al. 2000).

### Antibiofilm activities of designed peptides

#### Conventional CV method

There was 90 µL of 10^6^ CFU/mL MRSA in MHB media with fructose transferred into wells. Then, 10 µL of 1:2 serial dilutions of the antimicrobial peptides (1280–0 µg/mL) was added in wells. So that, final concentrations of peptides were 128–0 µg/mL. Plates were incubated at 37°C overnight. After overnight incubation, they were analyzed with conventional CV method. For this purpose, the bacterial cultures were removed, and wells were washed with distilled water. 200 µl of 1% CV dye was added to each well and incubated at room temperature for 15 min in the dark. Then, CV dye was removed, and wells were washed with water. After dried the plate in an oven at 45–50°C, 33% acetic acid was added into wells to dissolve the dye and wells were spectrophotometrically read by Varioscan (Thermo, USA) at 590 nm (Stepanović et al. 2000). Biofilm inhibiton rates (%) were calculated by the following formula (Liu et al. 2021):

$$(1-{{\text{A}}}_{590}\,\mathrm{ of\, wells\, treated\, with\, peptides}/{{\text{A}}}_{570}\,\mathrm{ of\, wells\, treated\, without\, peptides})\times 100$$

Before this calculation, the values obtained from all wells were subtracted from the value from the empty wells (Blank).

#### CoMIC method

Ninety microliters of 0.5 McF Methicillin-resistant* S. aureus* (MRSA) (1.5×10^8^ CFU/mL) in Congo Red Broth media with fructose was transferred into wells. Plates were incubated at 37°C. At the time just before the start of biofilm formation as optimized in Fig. 4, 10 µL of 1:2 serial dilutions of the antimicrobial peptides (2560–0 µg/mL) was added in wells. So that, final concentrations of peptides were 256–0 µg/mL. Incubation of media continued overnight. Then, wells were evaluated according to their black color formation.

#### SEM (scanning electron microscope)

The effect of NET1 peptides on the integrity of *E. coli* cell membranes was shown by SEM (Wang et al. 2018). 16 µg/mL was used as the peptide concentration, which is 8–16 times the MIC value. Peptide molecules were mixed with 10^5^ CFU/mL of *E. coli* and incubated at 37°C for 4–5 h. As suggested in the literature, the incubation time in this study was intentionally kept short due to the possibility of dissociation of bacteria combined with the peptide. After incubation, 20 µL of the mixture containing peptide antibiotics and bacteria was applied to a dialysis membrane. Then, it was fixed and imaged by the SEM microscope. As a negative control, bacteria which was not treated with peptide molecules were also used.

## Results

### Sequences and properties of designed antimicrobial peptides

In this study, four peptide sequences were designed by using D- and L-form amino acids. The properties of the peptides numbered as NET1, NET2, NET3, and NET4 shown in Table 1.

### Molecular modelling (docking and molecular dynamics) of the AMPs

The 3D structure of the L-amino acid forms of the peptides were obtained by PEP-FOLD3 (Thevenet et al. 2012), but the 3D structures of the D-amino acids could not do, since it cannot predict peptides with unusual L-amino acids or D-amino acids. Therefore, all the other 3D structures were created using tcl scripting in VMD since they include D- forms of peptides: as it is known, since the L and D form of amino acids are mirror images of each other, keeping the positions of the amino acids in the alpha helix of the peptides, only the direction of the side groups according to chirality center has changed.

**Fig. 1:**
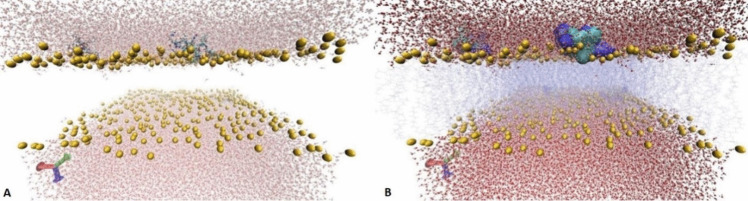
Molecular simulation with ~50 ns duration after positioning the NET1 peptide molecule on the bacterial membrane and in water, (**A**) side view, (**B**) view from a different angle. Water molecules and lipids are closed when visualizing, phosphate molecules are shown with yellow VDW balls, and peptides are shown with CPK atoms

With the first molecular modelling simulations, it was checked whether the peptides attached to each other in the water phase and remained in that phase without being directed to the membrane or not. In molecular modelling studies, four of the same peptides were placed in the water phase above the membrane. A simulation box of approximately 160×160×108 Angstroms^3^ was created similarly and modelled for each designed peptide (Fig. 1).

A total of ~50 ns MD simulation was performed using it as the initial input. This simulation result of the NET1 peptide is visualized in Fig. 1. During the simulation period, it was observed that the NET1 peptide molecules tended to be directed and adhered to the membrane.

In the 50 ns simulation, it was observed that the peptides started to attach to the membrane starting from about 15 ns and moved into the membrane and remained bound throughout the simulation.

### Chemical synthesis of AMPs and purifications with HPLC

In each synthesis, the amount of the amino acid used and the ml amount of DMF that was dissolved in were calculated by the program of the CEM Liberty Blue® peptide synthesizer, and their chemical synthesis was carried out.

**Fig. 2:**
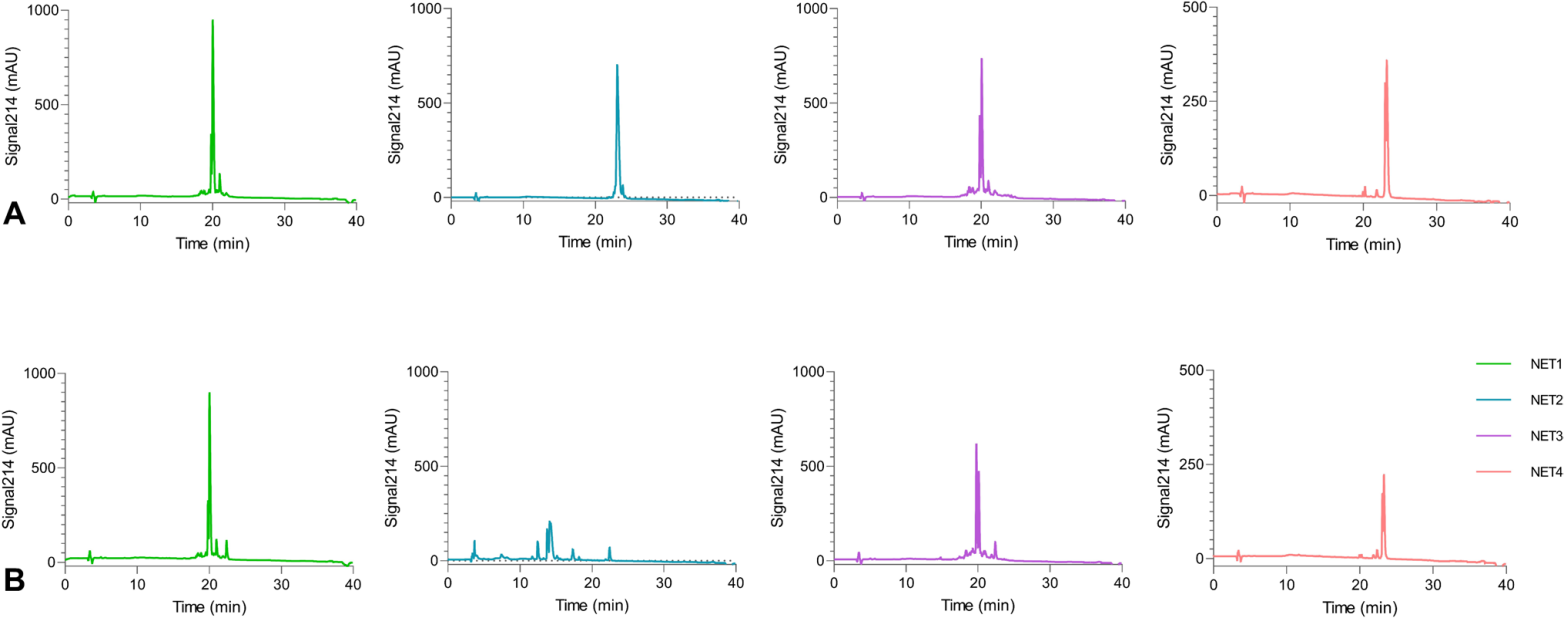
(**A**) HPLC chromatogram of peptides before proteinase K treatment (**B**) HPLC chromatograms of the peptides after treated with proteinase K, comparing the D and L forms

The peptides synthesized with the CEM Liberty Blue® peptide synthesizer obtained ~ 95% purity with a firm and device warranty. In the analytical HPLC results, a single peak was observed in the peptides (Fig. 2).

### Protease assays

Four peptides synthesized in D and L form were tested for their resistance to proteases. The analytical HPLC results of the peptides after being treated with proteinase K, comparing D and L forms, are as in Fig. 2. According to HPLC analysis, while NET2 peptide consisting of L form amino acids was degraded, other peptides showed resistance against proteases.

### Minimal inhibitor concentration (MIC) assay

MIC studies were performed with clinically important strains such as *E. coli* ATCC25922*, S. aureus* ATCC25923, MRSA, VRE, *E. coli* ER2566 and *E. coli* NTCC13846. including hospital infection, biofilm, and antibiotic resistance of our designed AMPs. MIC values are as in Table 2.

### Cytotoxicity and hemolytic activity

**Fig. 3:**
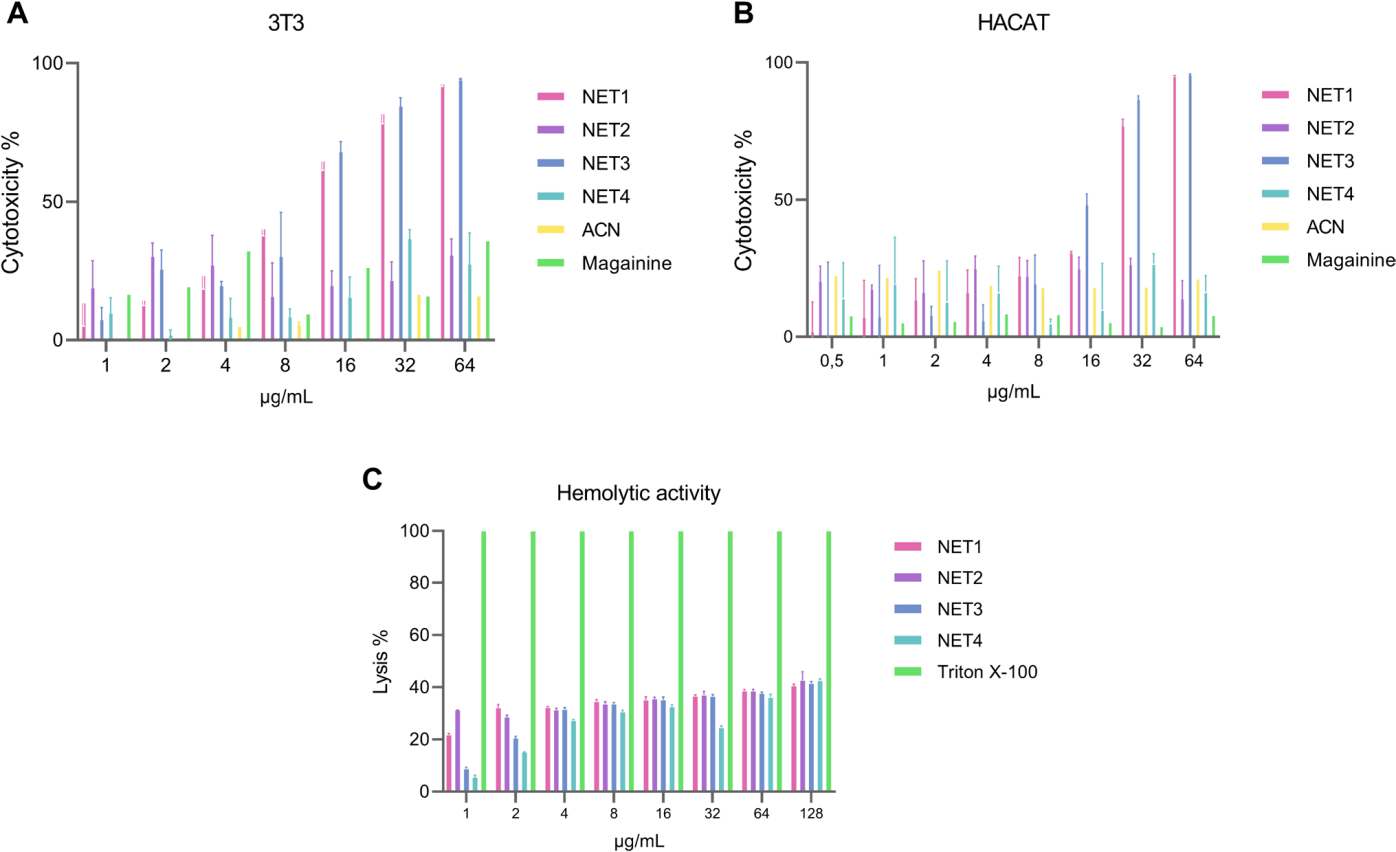
% Cytotoxicity rates for NET1, NET2, NET3, NET4, Magainin 2, and ACN in 3T3 cell line (**A**), in HaCaT cell line (**B**), NET1, NET2, NET3, NET4, and Triton X-100% lysis rates (**C**)

As a result of the experiments, it was calculated that the hemolytic activities of the peptides were below the HC_50_ value accepted in the literature (Tincho et al. 2020), and the MIC values were up to a minimum of 4 times. The toxic effects of NET1, NET2, NET3, and NET4 peptides were examined on two different cell lines. In line with these results, the peptides ranged from 0.5 to 64 µg/ml but did not exceed 50% in the 3T3 cell line, and NET4 in the HaCaT cell line increased the toxicity above 50% only at a concentration of 64 µg/ml, while the other 3 peptides remained in the safe range at all concentrations (Fig. 3). According to this, for hemolytic activities, therapeutic indexes of NET1 and NET3 were 32, while NET2 and NET4 were 1 and 4, respectively. For cytotoxic activities on HaCaT, therapeutic indexes of NET1 and NET3 were 32, whereas NET2 and NET4 were 2 and 8, respectively. On the other hand, it was 16 for NET1 or NET3, but 2 and 8 for NET2 and NET4, respectively on 3T3 cells. Statistical analysis was performed with A two-way ANOVA in GraphPad Prism software to calculate the statistical probability. Differences in the data were statistically significant since the *p* value was equal to or less than 0.0001.

### CoMIC method as antibiofilm activity assay

**Fig. 4:**
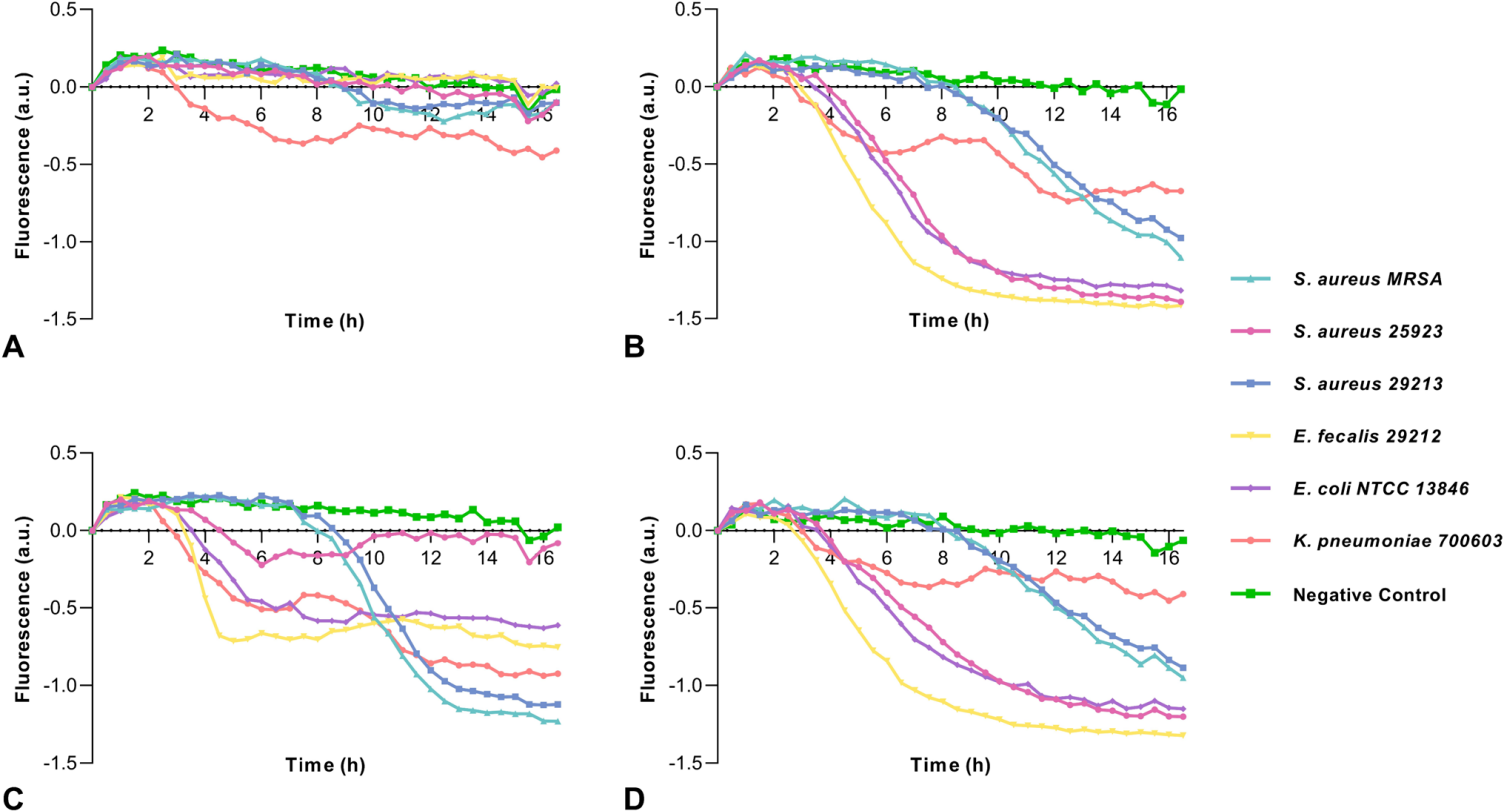
Fluorometric monitoring of biofilm production of bacteria in different sugar sources. Congro Red Broth without sugar (**A**), with glucose (**B**), with sucrose (**C**), and with fructose (**D**)

In the optimization of CoMIC method for real-time monitoring of color change due to biofilm formation, cultures of various biofilm-producing and non-biofilm-producing bacteria with different sugar sources were used as positive and negative controls for validation. Time-dependent fluorescence changes during bacterial culture were plotted. After cultured overnight, the end point black color changes in the wells were as in Figure S1. No color change observed when *S. aureus* ATCC 25923 were cultured in Congo Red Broth (CRB) only, but it was a return to all black color when added glucose and fructose into CRB culture. However, very little to no color change was observed in sucrose-containing medium. For the other two types of *S. aureus* such as *S. aureus* ATCC 29213 and *S. aureus* MRSA, color transformations occurred in all sugar-containing media but only showed a weak development in the sugar-free culture. *E. faecalis* ATCC 29212 and *E. coli* NTCC 13846 gave a similar pattern with different sugar sources in CRB. All sugar sources converted to black color, but not the one without sugar. *K. pneumonia* ATCC 700603 culture completely turned black when used sucrose, but the change was partial with glucose or without any sugar. There was no change when used fructose. *P. aeuroginosa* ATCC 27853 culture showed no color change at all because it is not a biofilm producer (Perez et al. 2011). This optimization study verified that the biofilm-associated color change of CRB was as expected when biofilm-producing and non-biofilm-producing bacteria were used. Graphics showing the real-time fluorescence changes of the overnight cultures were as in Fig. 4.

*K. pneumonia* ATCC 700603 culture without sugar showed little fluorescence change after 3 h later, whereas *S. aureus* ATCC 29213 and *S. aureus* MRSA did after 9 h later. Other bacterial cultures in CRB only did not show any fluorescence changes. When used glucose, in all bacterial cultures, except *P. aeuroginosa* ATCC 27853, fluorescence changes were observed, although the initial amount of bacteria was the same, time of the change was different: *K. pneumonia* ATCC 700603, *S. aureus* ATCC 25923, *E. faecalis* ATCC 29212, and *E. coli* NTCC 13846 were after 3–4^th^ hour, *S. aureus* ATCC 29213 and *S. aureus* MRSA were after 9^th^ h. There were fluorescence changes in all bacterial cultures including sucrose, except *S. aureus* ATCC 25923. Time of the change was the same for all of them. The results of the fructose-containing culture were the same as one with sucrose, except that a fluorescence change was observed in *S. aureus* ATCC 25923 at 4^th^ hour. The fluorescence change of *P. aeuroginosa* ATCC 27853 culture used as negative control was positively increased, which is related to the production of fluorescent pigment by this bacterium in MHB medium (Fig. S2). As expected, no fluorescence change was observed in negative control cultures without bacteria. All these results are consistent with the end point black color changes in the wells, except *K. pneumonia* ATCC 700603 culture including fructose. While no black color transformation was observed when evaluated with the naked eye, a signal change was detected as a result of fluorometric measurements. However, the change in fluorescence intensity is relatively low and irregular, not the steady curve of the others.

**Fig. 5:**
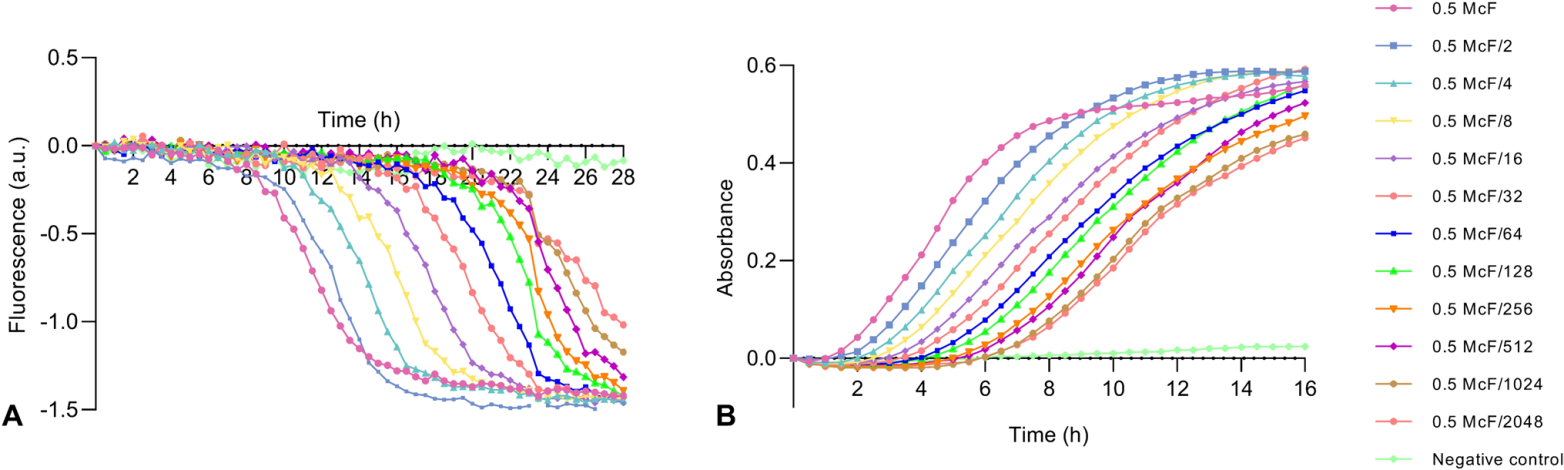
Fluorometric (**A**) and spectrophotometric (**B**) changes as a function of bacterial density

**Fig. 6:**
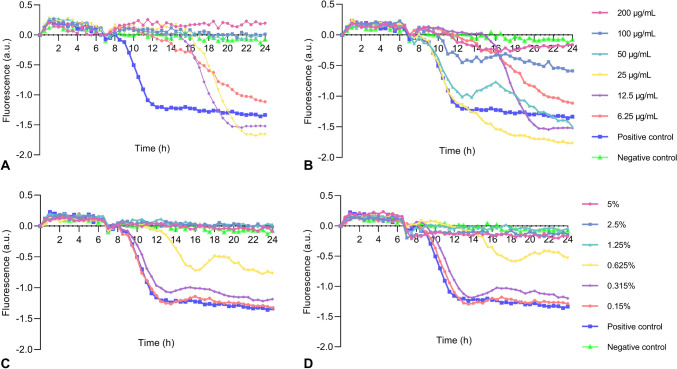
Real-time monitoring with CoMIC Method of antibiofilm activities. Ciprofloxacin added before (**A**) and after (**B**) the start of biofilm formation; sodium hypochlorite added before (**C**) and after (**D**) the start of biofilm formation

After verifying the real-time tracking of the color change related to biofilm production, the relationship between different numbers of bacteria and black color change was examined. For this purpose, serial dilutions of 1/2 between 0.5 McF and 0 bacteria were added to CRB medium, and the fluorescence changes were plotted against time (Fig. 5A). The growth of bacteria was also read spectrophotometrically at 600 nm (Fig. 5B). The results obtained proved that there was an exact correlation between turning the color of the medium black and the bacterial concentration (Fig. S3). This made it possible to determine when biofilm formation started. After establishing this, the effects of chemical compounds to the CoMIC medium just before the beginning of biofilm formation were investigated to determine the minimal inhibition concentrations for biofilms. For this purpose, the effects of different concentrations of ciprofloxacin and sodium hypochlorite on the biofilm formation of *S. aureus* 29213 were tested and fluorescence changes were measured (Fig. 6). The same conditions were also performed and analyzed with standard CV method as a replica 96 well plate for comparison (Fig. S4).

When studied using the approach practiced in the literature which is based on the principle of adding chemicals at the very beginning of the biofilm culture, MIC for biofilms values were as follows: for ciprofloxacin and sodium hypochlorite, it was 50 µg/mL and 1.25% respectively. However, based on the fluorescence change of CoMIC method, when chemicals were added just before the start of biofilm formation, MIC values for biofilms were >200 µg/mL and 1.25% for ciprofloxacin and sodium hypochlorite, respectively. Plots of the time-dependent change in fluorescence signal are consistent with the endpoint black color change image in 96-well plates (Fig. 6 and Fig. S4).

In CV method, when chemicals are added in the very beginning, MIC values for biofilms were 12.5 µg/mL and 1.25% ciprofloxacin and sodium hypochlorite, respectively. When added just before biofilm formation, they were >200 µg/mL and 5%.

Subsequent to the proof of principle that this method can be used for antibiofilm activity assays, antibiofilm assays were performed for the peptides developed in this study using both this method and the classical CV method. For this purpose, peptides were added into culture media just before the beginning of biofilm formation for CoMIC method, whereas it was added in the very beginning as in the literature. *MIC* results for biofilm *formation of* Methicillin-resistant *S. aureus* (MRSA) based on crystal violet (CV) method and CoMIC method were as in Table 3 and Figure S5. According to CV results, 100% biofilm inhibition rate is 4 µg/mL for NET1 and NET3; 128 µg/mL for NET2 and NET 4, respectively. When applied CoMIC method, 64 and 128 µg/mL for NET1 and NET3; >256 µg/mL for NET2 and NET4 were required for complete inhibition of the biofilm.

**Table 3:** Peptide concentrations (µg/mL) inhibiting the biofilm at 100%, which obtained by the CV method and the CoMIC method

(µg/mL)	NET1		NET2		NET3		NET4	
	Crystal Violet	CoMIC	Crystal Violet	CoMIC	Crystal Violet	CoMIC	Crystal Violet	CoMIC
*MRSA*	4	64	128	>256	4	128	128	>256

According to the results obtained by the CV method, NET1 and NET3 peptides can show anti-biofilm activity at lower concentrations than NET2 and NET4 peptides. These sequences, in which D and L form amino acids are used together, show better biofilm inhibition activity.

In addition to the biofilm inhibition experiment with CV, the CoMIC method, which is based on the starting point of the biofilm, was designed, and performed here. Since biofilm formation depends on the increase in bacterial concentration and the accumulation of quorum sensing metabolites that enable communication between bacteria, the use of Congo red, which is characterized by the formation of a black pigment with the biofilm, was investigated to determine exactly when biofilm formation starts. In order to obtain a real-time observable growth curve of the black pigment production of the biofilm depending on the bacterial concentration, biofilm culture was prepared by adding different numbers of bacteria to broth media containing Congo red in 96-well plates; biofilm formation was observed in a spectrophotometer in a time-dependent manner. After this optimization, by using the CoMIC method, peptides were added to biofilm culture media once the color of the culture media turns to black depending on biofilm formation, it was aimed to find the actual biofilm inhibiting concentration. Looking at Table 3, the biofilm inhibition values obtained by the CoMIC method were higher than the values obtained by the CV method, because peptides were added when the bacteria reached a higher concentration at which they started to biofilm formation. According to the data we obtained with this method we developed, NET1 and NET3 peptides have a biofilm inhibition effect at lower concentrations than the other two peptides.

### Scanning electron microscopy (SEM)

**Fig. 7:**
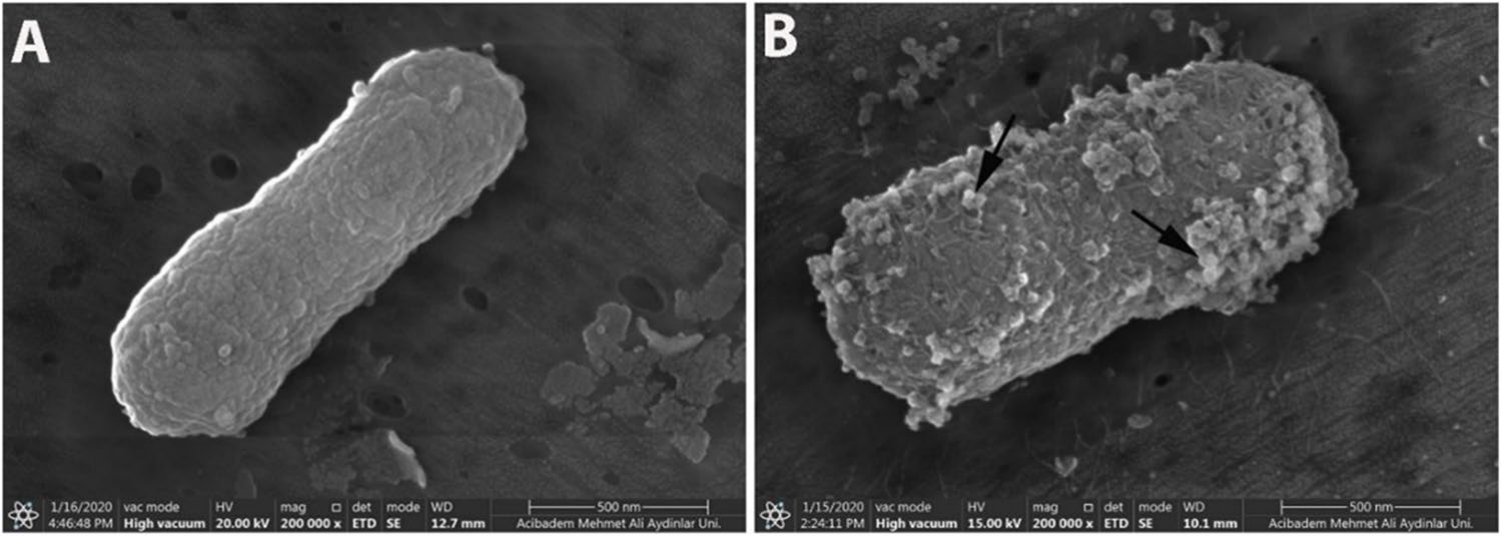
SEM images of *E. coli* bacteria. The control group showed smoother cell surface morphology (**A**), and numerous small bubbles (arrow) were observed after treated with the NET1 peptide (**B**)

Examination of the images taken by SEM revealed that the *E. coli* used as controls had smoother cell surface morphology than those treated with NET1 peptide (Fig. 7). It was observed that different shapes of blisters and numerous small bubbles were seen in the *E. coli* treated with peptides. NET1 peptide has been shown to be lethal to *E. coli*.

## Discussion

Antimicrobial resistance has been a serious problem affecting all living organisms from past to present. Among the causes of this problem is unconscious and excessive use of antibiotics. The increase of pollution in nature with the increasing population causes mutations in microorganisms. All these events lead to the formation and progression of antimicrobial resistance. One of solutions is the development of new antibiotics and AMPs are considered highly promising for that, which can be found naturally in the immune systems of living organisms, or designed and synthesized synthetically.

Self-interacting peptides often contain ionic and hydrophobic groups in their structures. In antimicrobial peptides, positively charged cationic amino acids such as R or K are preferred as counterparts to ionic amino acids (McCloskey et al. 2014), which maximizes peptide membrane interaction. Besides the properties of the peptide, the ionic strength of the solvent and the peptide concentration also affects it. Based on this information, we have preferred positively charged cationic amino acids in the design of our peptides.

In this study, we aimed to design peptides with high antimicrobial activity that are effective against biofilms formed by *S. aureus* MRSA, a serious problem in hospitals. Considering the importance of proteases in the formation of biofilms, it was also aimed to be resistant to protease enzymes of the developed peptides. Since it is well-known that D amino acids are resistant to proteases but are also toxic to cells, we designed peptides containing both L- and D-form amino acids at the same time that had protease resistance with low toxicity (NET1-NET3). We also synthesized the peptide entirely composed of the L-form (NET2) and the D-form (NET4) to compare the toxic effect and protease resistance. As a result, the NET2 peptide (containing the L form amino acid) was completely degraded, but NET1, NET3, and NET4 peptides (containing at least one D form amino acid) were not fractionated by proteases, which they have been shown by analytical HPLC analysis. Designed peptides were subjected to molecular modeling of membrane interaction; antimicrobial activity, biofilm inhibition, hemolytic activity, cytotoxic activity assays; Scanning Electron Microscopy (SEM) analysis.

According to MIC results, NET1 and NET3 peptides (containing the D and L amino acid forms) showed antibacterial activity at low concentrations, while NET2 and NET4 peptides (consisting entirely of L-form and D-form, respectively) had antibacterial activity at higher concentrations. Based on these results, it is clear that the combination of D- and L-form amino acids in the peptide structure greatly increased the antibacterial effect. It might be because the conformational change in the structure of the peptides depending on D- or L- forms of amino acids contributes to the increase of the antibacterial effect. Since the peptides designed here as α-helical contain amino acids in different forms, their branches have changed direction, and this has increased the impact area of the peptides. Accordingly, it was observed that the antibacterial effect of the peptide increased 8–16 times compared to the peptide containing D-form amino acid and increased 16–32 times compared to the peptide containing L-form amino acid. In the study by Manabe and Kawasaki (2017), the peptide consisting of D-form amino acids had 16 times better activity in *S. aureus* and 2 times better in *E. coli*. In another study (Dean et al. 2011), only the L- and only D-form of LL-37 peptide were reported that the activity of the D-form was 10 times lower than L-form. As a result, our peptides designed in this study exhibit similar or better antibacterial activity compared to antibiotics in the literature (Rima et al. 2021).

According to molecular modelling studies, NET1 peptide molecules tends to move towards and adhere to the membrane within nanoseconds. When treated bacteria with NET1 peptide molecules for 3 h, differences in the morphological structure of the bacteria were microscopically imaged by SEM.

Based on hemolytic activity assays, the HC_50_ value of four peptides was found as 64 µg/mL. Since the HC_50_ value was 16–32 times the MIC value of the NET1 and NET3 peptides, the concentrations of the peptides at which they showed antibacterial activity are in the safe range. Also, HC_50_ value for the NET2 peptide was 2–8 times the MIC value, which is in the safe range. However, the MIC value for NET4 peptide was determined as 64 µg/mL in 4 out of 5 bacteria, which means is not in the safe range.

The cytotoxicity values of the peptides were tested in two different cell lines. It is desirable that the IC_50_ value is high in healthy cell lines that is, the peptide does not produce cytotoxic effects even at high concentrations. The IC_50_ values of NET1 and NET3 peptides in HaCaT and 3T3 cell lines are 64 µg/mL and 32 µg/mL, which means 16 to 32 times the MIC values for NET1 and NET3 peptides, respectively. So our peptides are in the safe range in both cell lines. The IC_50_ value of the NET2 peptide is >128 µg/mL in HaCaT and 3T3 cell lines. It is seen that >2-4 times of MIC value does not affect cytotoxicity. The NET4 peptide has >128µg IC_50_ value of on 3T3 and HaCaT cell lines. The NET4 peptide did not show cytotoxic effects in any of the 2 cell lines at concentrations between 4 and 16 times the MIC value. The IC_50_ value of the NET2 and NET4 peptides were >128 µg/mL in HaCaT and 3T3 cell lines, which means >2–4 times or 4 and 16 times of MIC values. Therefore, they do not show cytotoxic effects too.

Effects on biofilm inhibition of designed peptides were also investigated. In the conventional biofilm inhibition assays (Cruz et al. 2018), peptides are added to biofilm culture media at the very beginning. Since it looks like MIC assays, minimal biofilm inhibition concentration results and MIC results in this study were close to each other as expected (Cruz et al. 2018).

By using the CV method, 100% biofilm inhibition was achieved with 4 µg/mL for NET1 and NET3; 128 µg/mL for NET2 and NET 4, respectively. Since the deviation in the non-specific binding of crystal violet dye is about 16.7 (Figure S6), 64 µg/mL of NET4 and concentrations less than 1 µg/mL of NET3 could be enough to inhibit the biofilms completely. Also, 2 µg/mL of NET1 and 64 µg/mL of NET2 provided over 50% inhibition. When applied CoMIC method, 64 and 128 µg/mLfor NET1 and NET3; >256 µg/mLfor NET2 and NET4 were required for complete inhibition of the biofilm. Previous studies with different bacteria showed that the concentration of chemicals required for biofilm inhibition were between 1.25 and 150.000 µg/mL (Roy et al. 2018). Compared to the literature, it is clear that our peptides also have a very high antibiofilm activity when tested the conventional methods (Thieme et al. 2019).

Since bacteria are introduced to antimicrobial agents long before they begin to form biofilms, this conventional approach (Cruz et al. 2018) is more similar to the minimum inhibitor concentration (MIC) method than the minimum biofilm inhibitor concentration method. For this reason, MIC results and antibiofilm results are very close in the literature (Cruz et al. 2018), and when we repeated this method, our results are also consistent with the literature (Cruz et al. 2018) as explained above. Unfortunately, the biggest challenge in testing new anti-biofilm agents is the lack of a reliable and accurate method for their screening (Cruz et al. 2018; Thieme et al. 2019). These limitations of the current methods used in the literature and the suboptimal results obtained may also be impairing the prevention of biofilms in the clinic, which may be one of the reasons for the increasing problem of biofilms in the community. How can there be a more accurate innovative approach? The first logical solution to the problem is to monitor biofilm formation in real time and add antimicrobial agents as soon as biofilm formation starts. In this study, we investigated what kind of technique would make this possible and developed the CoMIC method. In summary, the CoMIC method was designed as a liquid culture medium containing Congo red and is based on real-time observation of the black color due to metabolites produced during biofilm formation. 1:100 dilution of fresh cultured bacteria are allowed to biofilm culture in liquid Congo red medium and antimicrobial agents are added at the point of black coloration, which represents the moment when biofilm formation begins. According to the results obtained with this method, the antimicrobial agent requirement for biofilm inhibition is 2–32 times higher than the classical method. This is understandable because the bacteria must first reach a certain number to form a biofilm, which is much above the number at the beginning of the biofilm culture as in the classical method, and therefore the amount required to inhibit biofilm formation is much higher. However, in the classical method, since it is impossible to know when the biofilm starts, antimicrobial agents are added to the medium at the very beginning of the culture and the MIC result is expressed as the antibiofilm result. The CoMIC method solves this problem and can detect the onset of biofilm formation as a color change, making it possible to add antimicrobial agents at the right stage. This observable method overcomes the limitations of classical methods by providing a new model for antibiofilm measurements that better simulates the real situation.

The black color change related to biofilm formation could be monitored in real time as proportional to the bacterial concentration. The graphic obtained is capable of generating quantitative data such as qPCR results. If the turbidity measurement in broth culture media without Congo Red as a replica was monitored in real time based on bacterial concentration, it became possible to estimate the number of bacteria required for biofilm formation. Accordingly, while the growth curve of the bacteria started to be graphed at second hour, it was seen that the fluorescence intensity change based on biofilm production of the same bacterial density began at the 8^th^ hour. *S. aureus* is a fast-growing bacterium, doubling in size every 20 min (Missiakas and Schneewind 2013). In this case, when the culture starting at 1.5×10^8^ CFU/mL reaches approximately 9×10^8^ CFU/mL, the turbidity-dependent growth curve was obtained, while the signal change due to biofilm production could be generated only when the bacterial density reached 27×10^8^ CFU/mL, which indicates the number of bacteria required for biofilm production. As a result, the CoMIC method provides a very important innovation in understanding biofilms and conducting the necessary quantitative studies. In conclusion, peptides with high antimicrobial activity were developed, which can be considered as promising candidates to fill the lack of suitable drugs against various microorganisms. A novel real-time detection method for antibiofilm activity assays has also been developed, named CoMIC.

## Supplementary Information

Below is the link to the electronic supplementary material.

Supplementary file1 (PDF 815 KB)

## Data Availability

All data generated or analyzed during this study are included in this article (and its supplementary information files).
