# Supplementary material for: New-generation biofilm effective antimicrobial peptides and a real-time anti-biofilm activity assay: CoMIC
Source: Appl Microbiol Biotechnol. 2024 May 3;108:316. doi: 10.1007/s00253-024-13134-1 (PMC11068671; doi:10.1007/s00253-024-13134-1)
Supplement: Supplementary file 1 [file 253_2024_13134_MOESM1_ESM.pdf]

Title Page

New Generation Biofilm Effective Antimicrobial Peptides and A Real-Time Anti-biofilm Activity Assay: CoMIC

Tuba Polat[1], İrem Soyhan[1], Sinan Cebeci[1], Tuğba Arzu Özal İldeniz[2], Özgül Gök[2], Merve Açıkel Elmas[4], Erkan Mozioğlu[1*], Nihan Ünübol[3*]

[1] Department of Medical Biotechnology, Institute of Health Sciences, Acibadem Mehmet Ali Aydinlar University, Istanbul, Turkey. [2] Department of Biomedical Engineering, Faculty of Engineering and Natural Sciences, Acibadem Mehmet Ali Aydinlar University, Istanbul, Turkey. [3] Department of Medical Microbiology, School of Medicine, Acibadem Mehmet Ali Aydinlar University, Istanbul, Turkey, [4] Department of Histology and Embriology, School of Medicine, Acibadem Mehmet Ali Aydinlar University, Istanbul, Turkey. [5]Medical Laboratory Techniques, Vocational School of Health Services, Acibadem Mehmet Ali Aydinlar University, Istanbul, Turkey.

*Email: nihan.unubol@acibadem.edu.tr; erkan.mozioglu@acibadem.edu.tr

Supporting Information

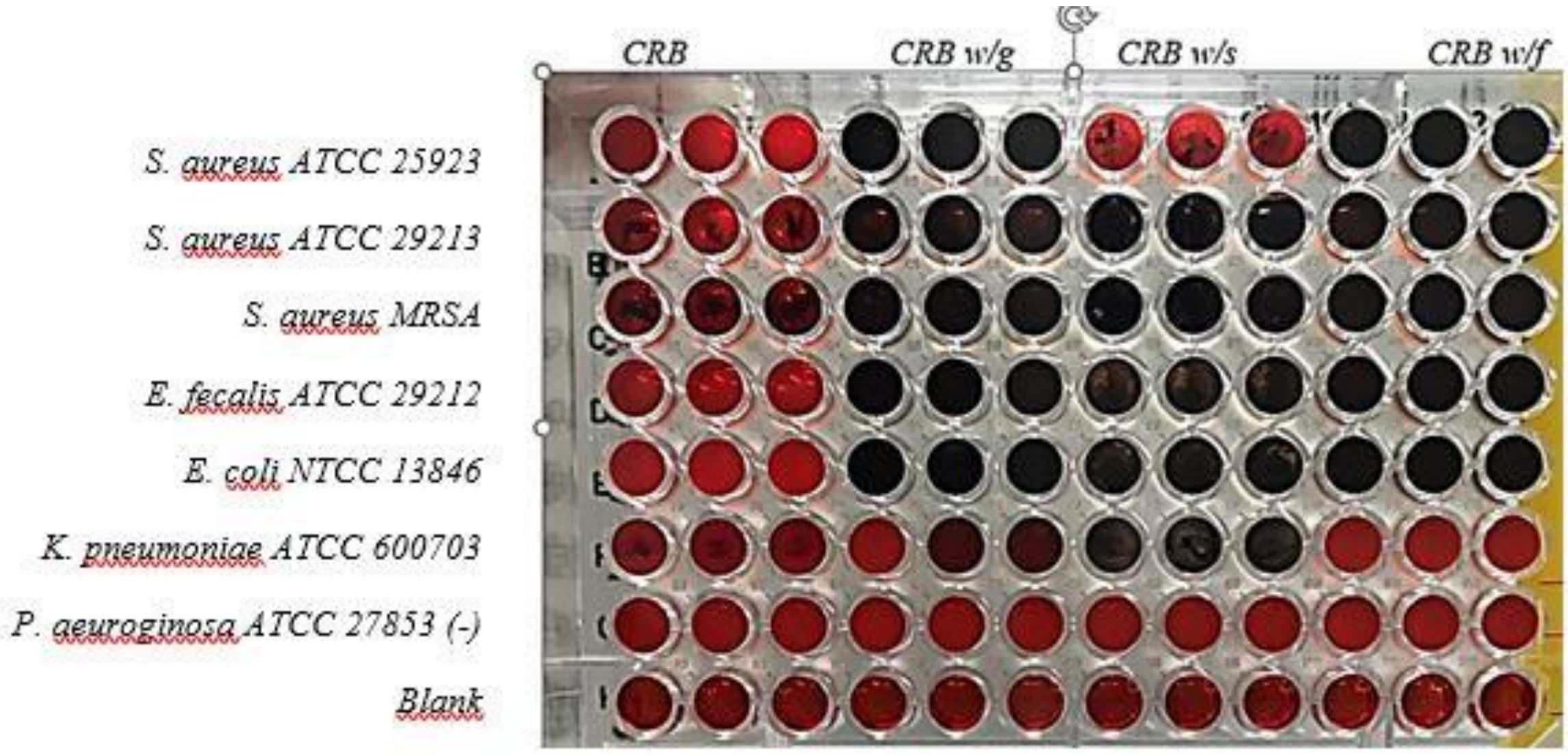

Fig S1. Biofilm production of bacteria in Congo Red Broth without sugar (CRB), with glucose (CRB w/g), with sucrose (CRB w/s), and with fructose (CRB w/f). Blank does not included bacteria.

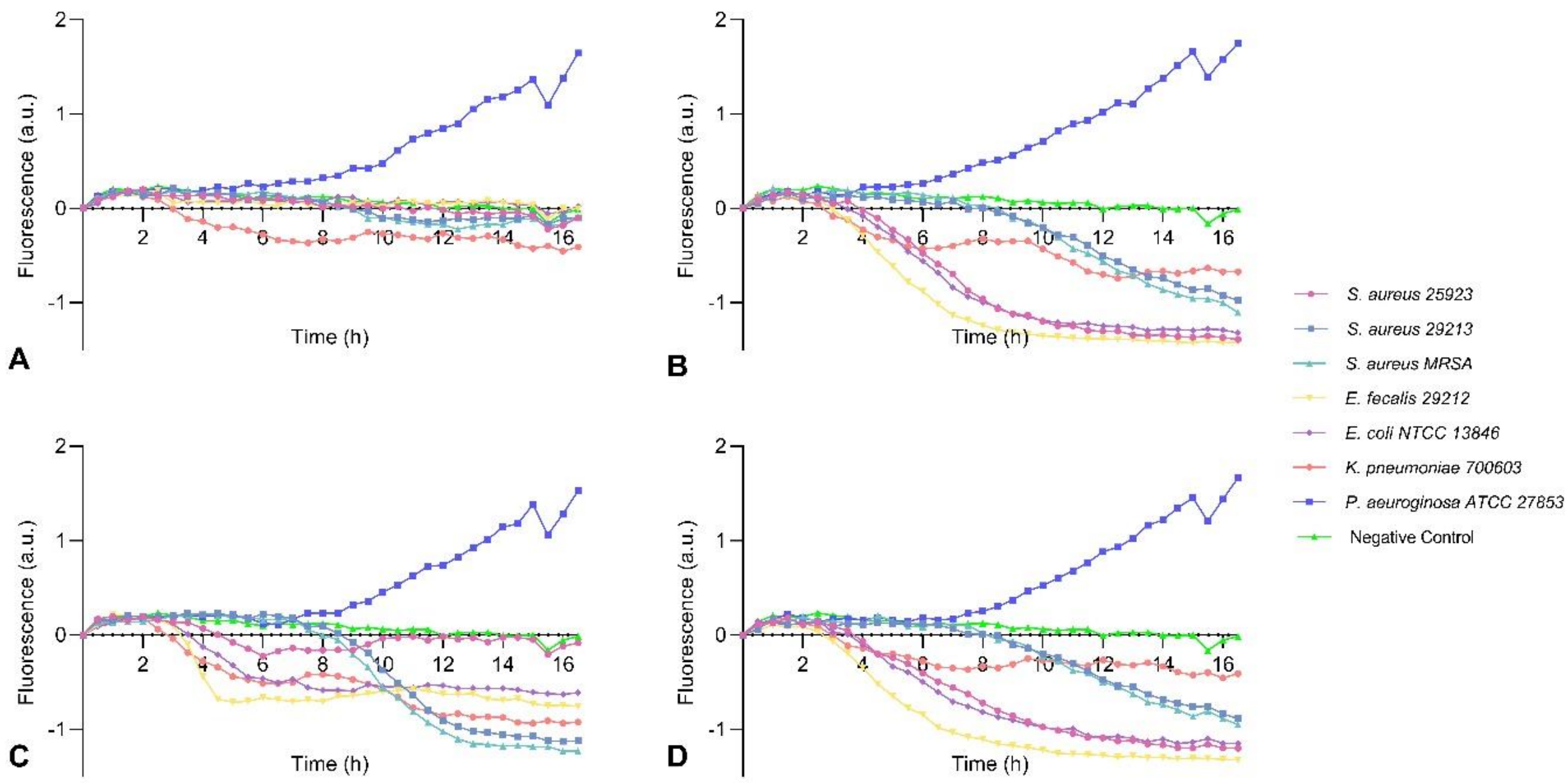

Fig S2. Fluorometric monitoring of biofilm production of bacteria in different sugar sources. Congro Red Broth without sugar (A), with glucose (B), with sucrose (C), and with fructose (D). The fluorescence change of *P. aeuroginosa* ATCC 27853 culture used as negative control was positively increased, which is related to the production of fluorescent pigment by this bacterium in MHB medium.

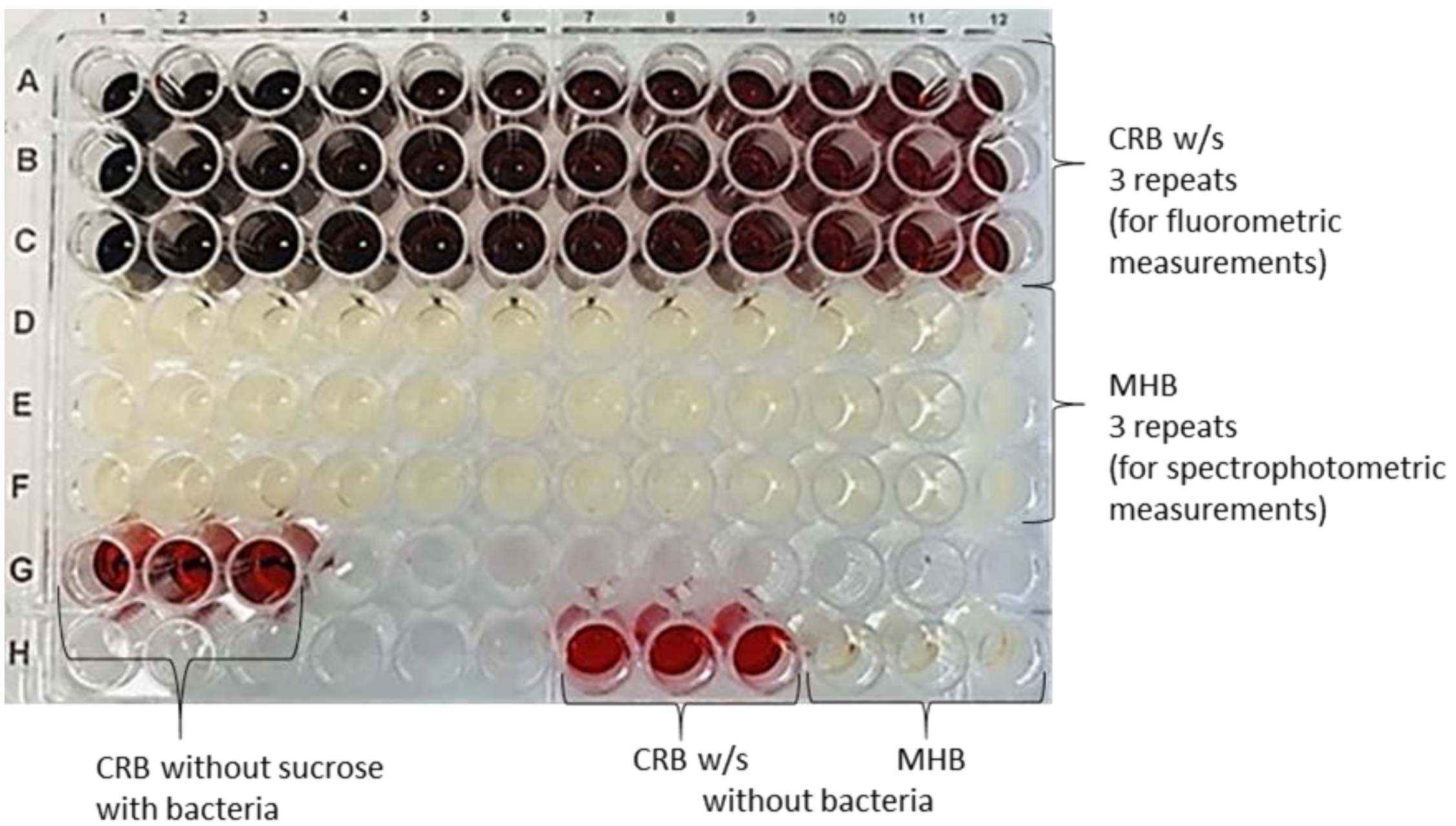

Fig S3. The 96 well plate used for measurements of fluorometric (A) and spectrophotometric (B) changes as a function of bacterial density.

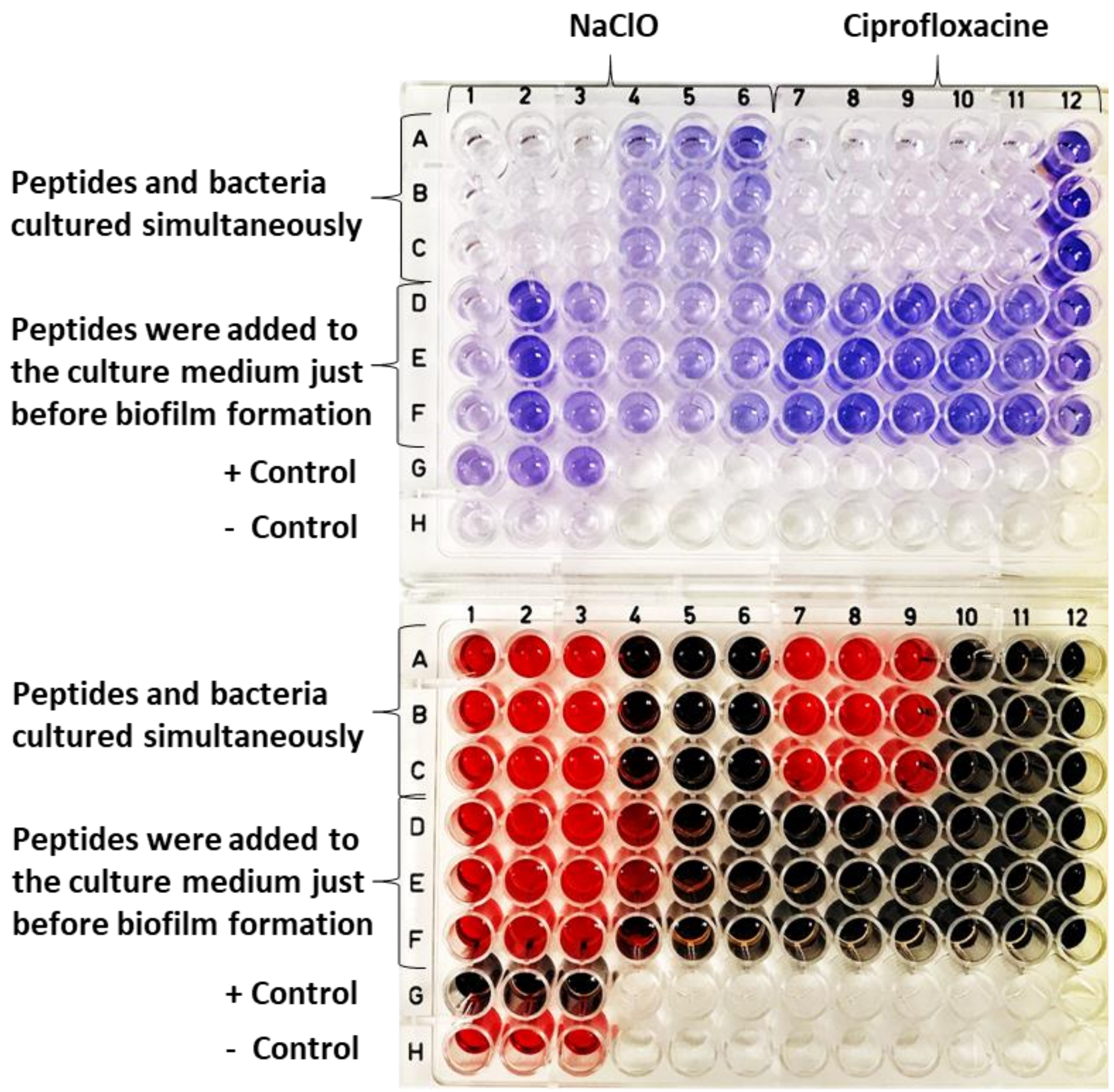

Fig S4. The 96 well plates used for CV method (top) and real time monitoring with CoMIC Method (bottom) of antibiofilm activities against sodium hypochlorite (left) and ciprofloxacine (right).

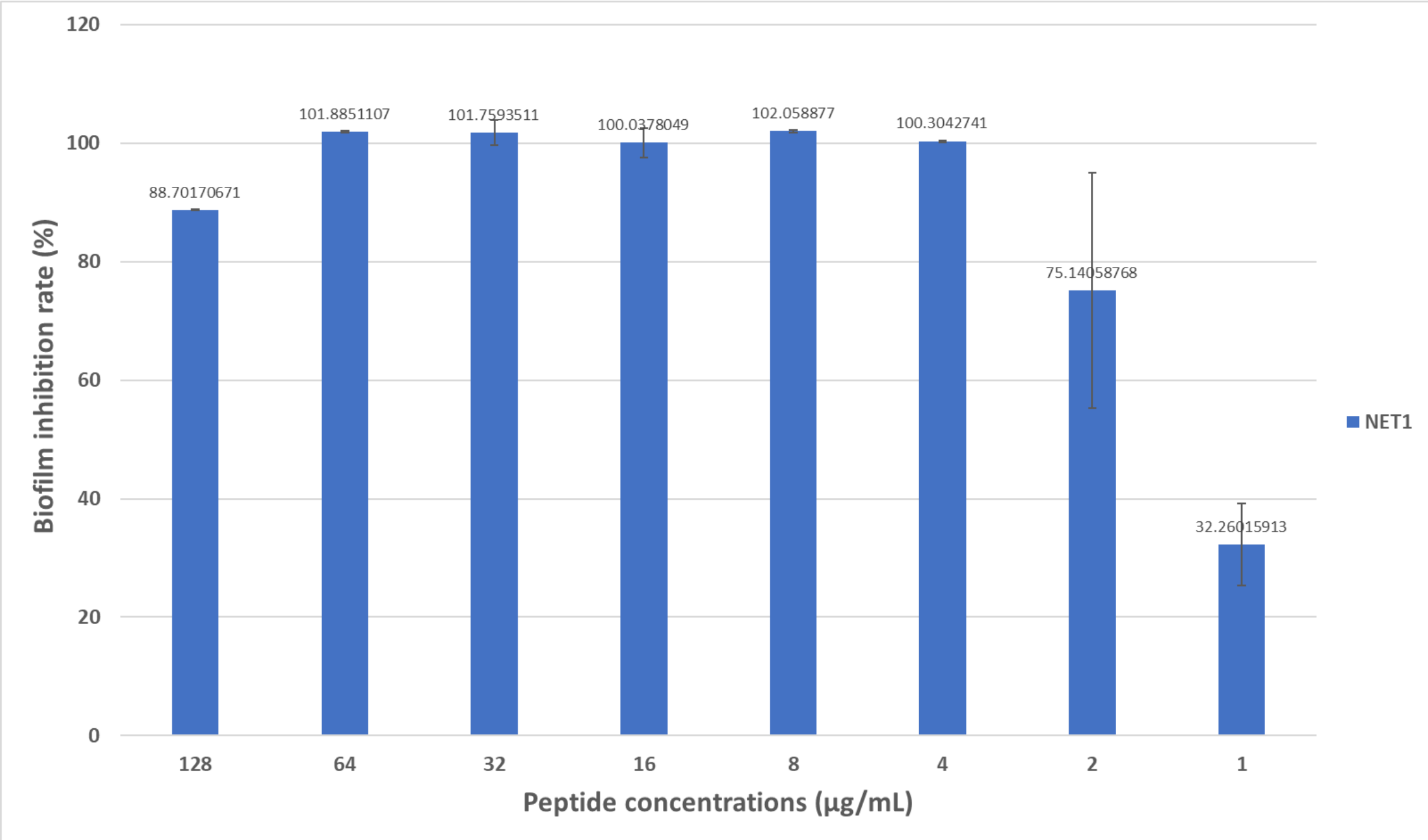
Biofilm inhibition rate (%)
120
100
80
60
40
20
0
88.70170671
101.8851107
101.7593511
100.0378049
102.058877
100.3042741
75.14058768
32.26015913
128
64
32
16
8
4
2
1
Peptide concentrations (µg/mL)
NET1

A

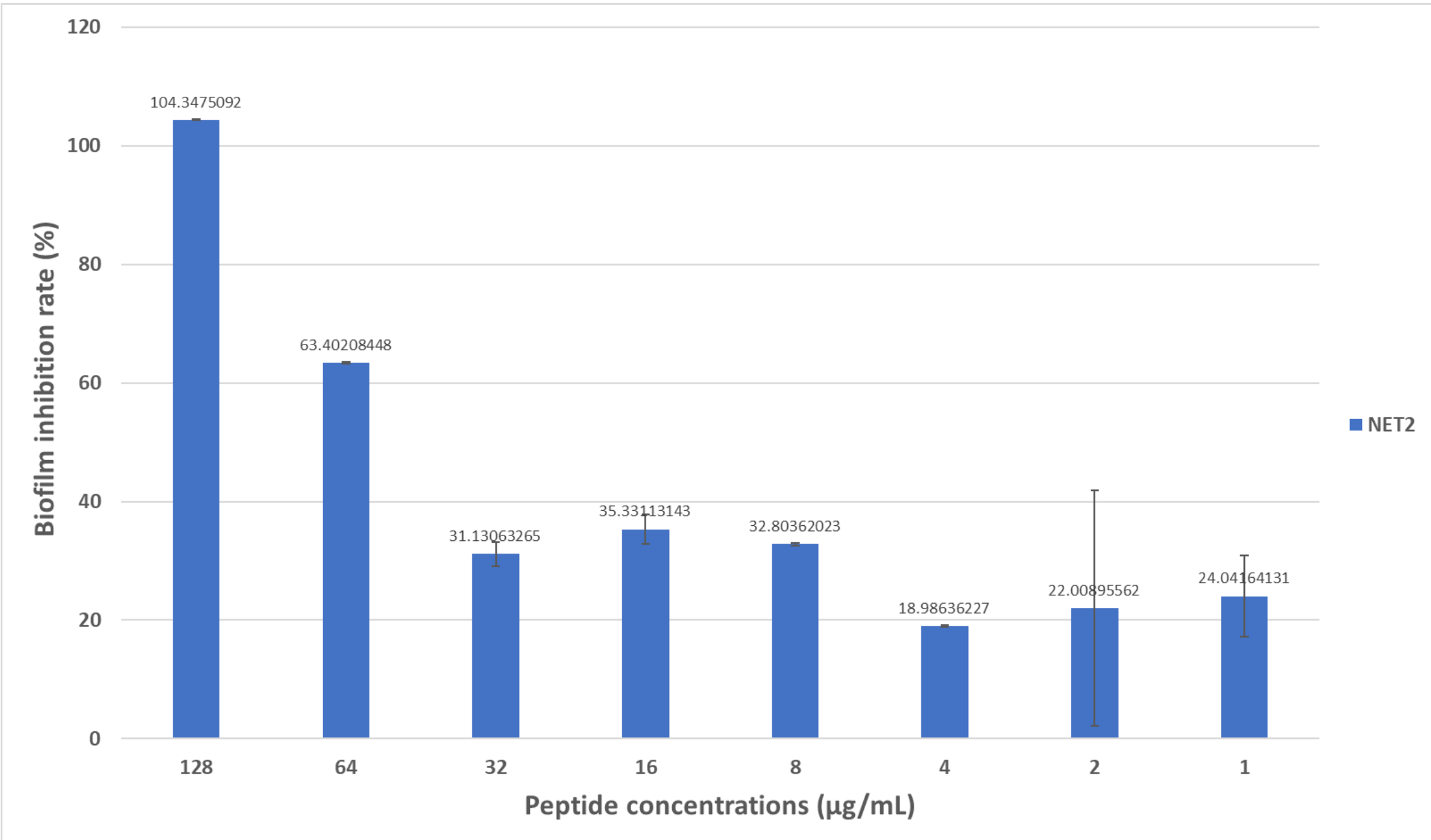
Biofilm inhibition rate (%)
120
100
80
60
40
20
0
104.3475092
63.40208448
31.13063265
35.33113143
32.80362023
18.98636227
22.00895562
24.04164131
128
64
32
16
8
4
2
1
Peptide concentrations (µg/mL)
NET2

B

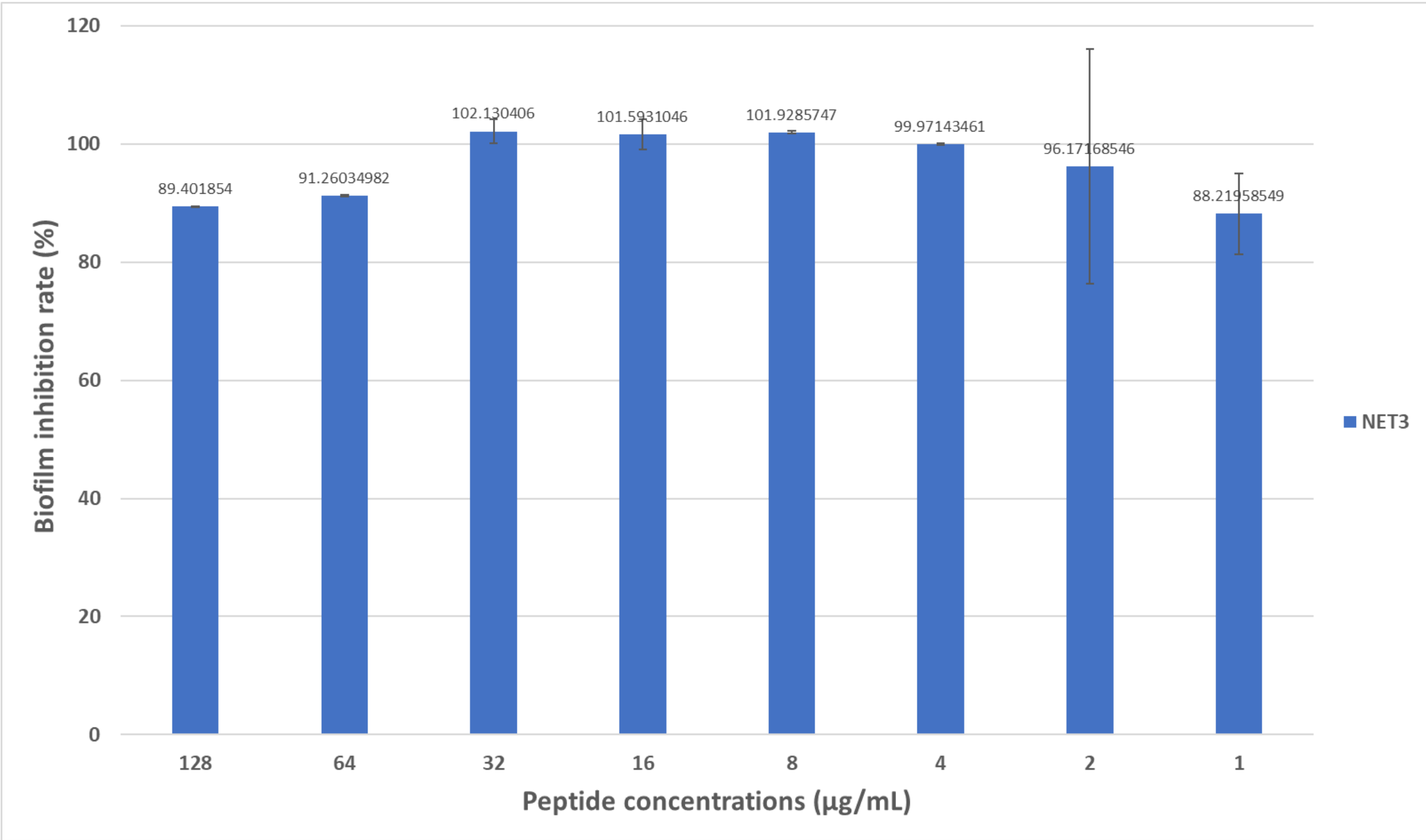

C

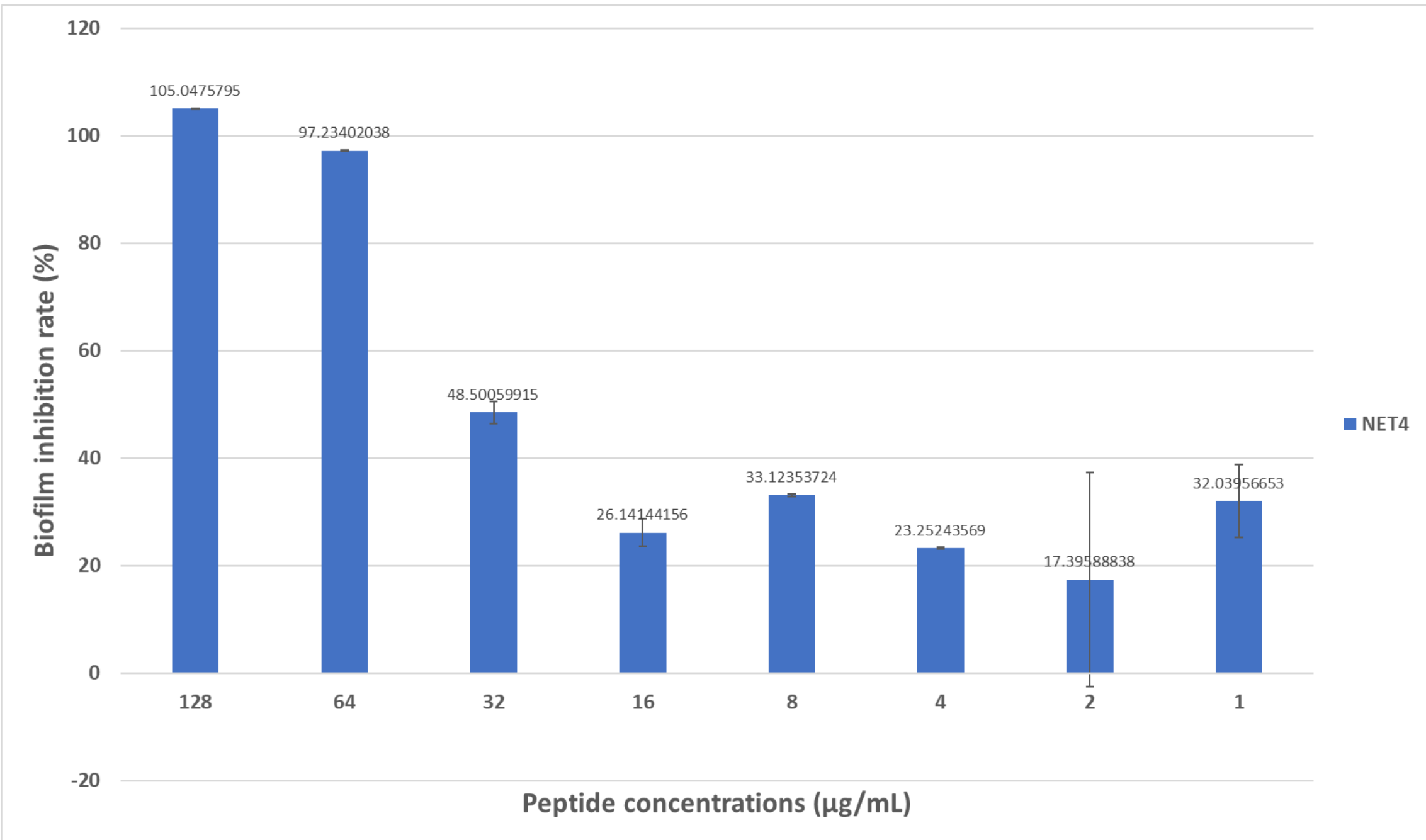

D

Fig S5. Biofilm inhibition rate (%) of NET1 (A), NET2 (B), NET3 (C), and NET4 (D) against MRSA strain with crystal violet method.

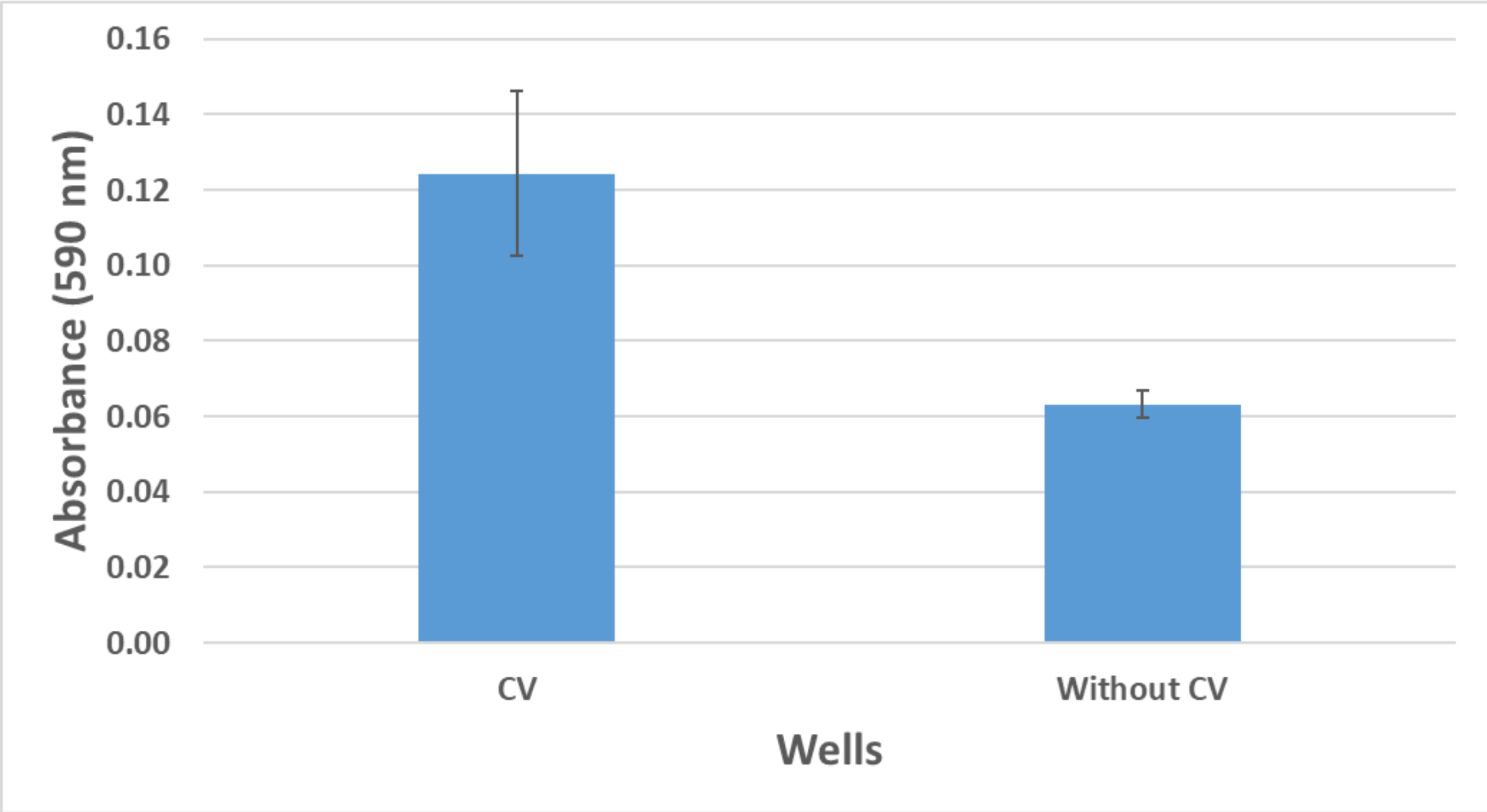
0.16
0.14
0.12
0.10
0.08
0.06
0.04
0.02
0.00
Absorbance (590 nm)
CV
Without CV
Wells

A

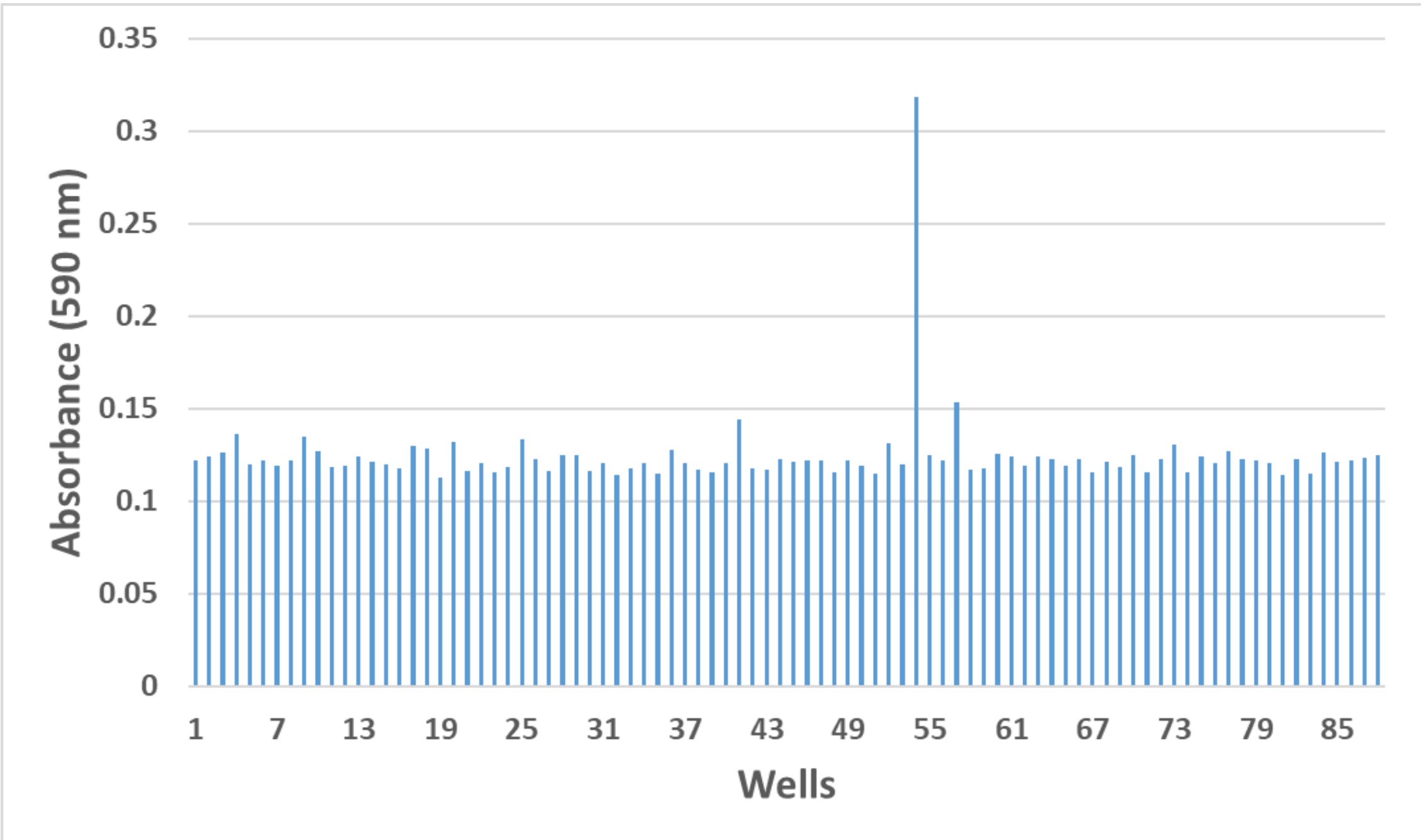
0.35
0.3
0.25
0.2
0.15
0.1
0.05
0
Absorbance (590 nm)
1
7
13
19
25
31
37
43
49
55
61
67
73
79
85
Wells

B

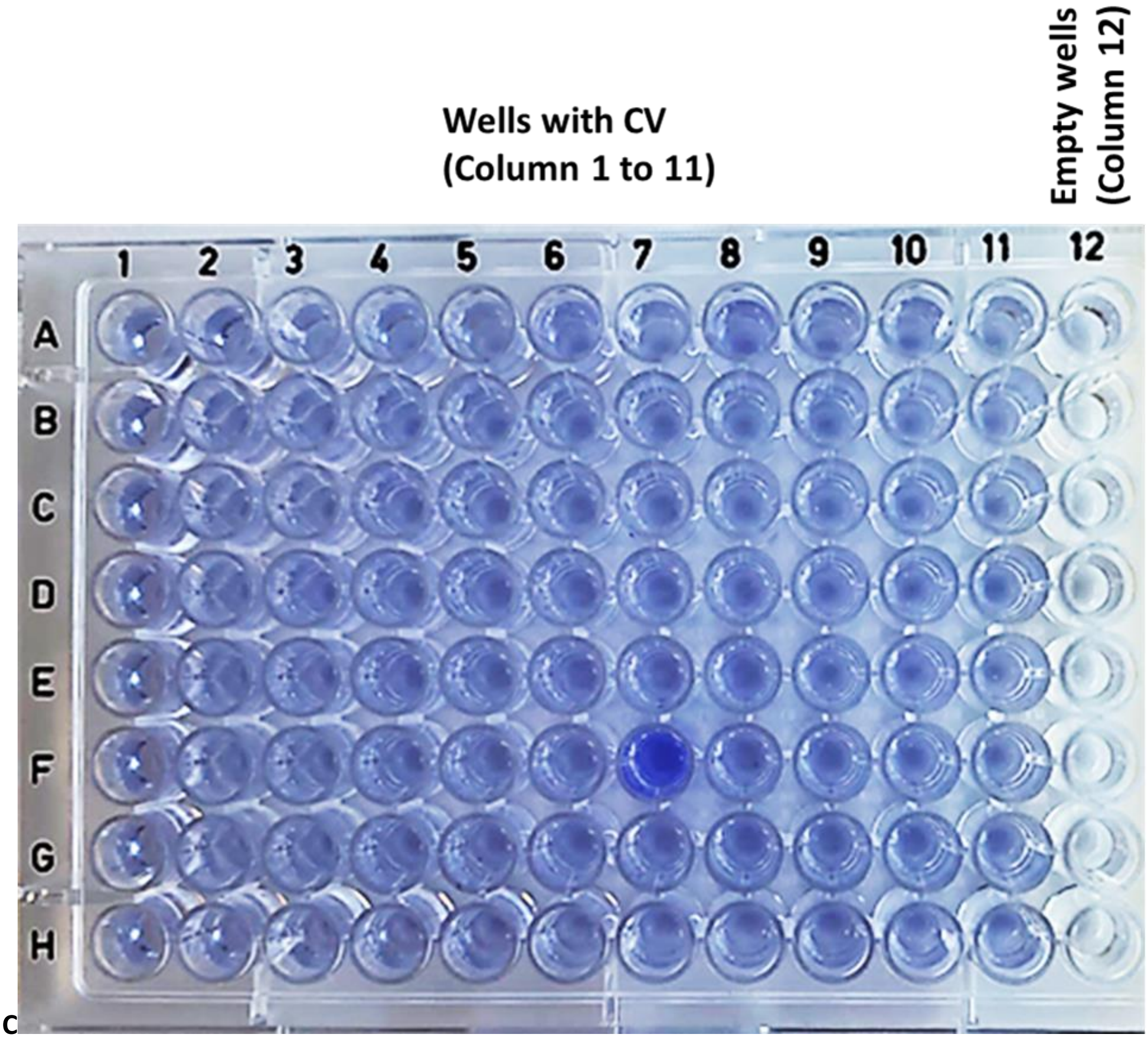

C

Fig S6. Standard deviation of measurements depending on Crystal Violet (CV). Absorbance values from wells with CV and without CV (A); individual absorbance measurement result of 88 wells (B). Image of the plate used for absorbance measurement (C).

These experiments were done under Conventional CV Method, except bacteria. Briefly, 200 µl of 1 % CV dye was added to 88 wells and incubated at room temperature for 15 min in the dark. Then CV dye was removed, and wells were washed with water. After dried the plate in an oven at 45-50 °C, 33% acetic acid was added into wells to dissolve the dye and wells were spectrophotometrically read by Varioscan (Thermo, USA) at 590 nm. As negative control, distilled water was used instead of the CV in 8 wells. Means and standard deviations were calculated. As a result of the non-specific binding of the crystal violet, approximately 2 times higher absorbance values are obtained. The deviation in the non-specific binding of crystal violet dye is about 16.7.
